# Adaptation differences and mechanisms of influenza viruses to ANP32 proteins across species

**DOI:** 10.1128/jvi.01900-25

**Published:** 2026-01-05

**Authors:** Zhenwei Bi

**Affiliations:** 1 Institute of Veterinary Medicine, Key Laboratory of Veterinary Biological Engineering and Technology, Ministry of Agriculture and Rural Affairs, Jiangsu Academy of Agricultural Sciences 668638 , Nanjing, Jiangsu, China; 2 Jiangsu Key Laboratory of Zoonosis, Yangzhou University 38043, Yangzhou, China; 3 GuoTai (Taizhou) Center of Technology Innovation for Veterinary Biologicals, Taizhou, Jiangsu, China; Indiana University Bloomington, Bloomington, Indiana, USA

**Keywords:** influenza, ANP32, interaction, adaptive mutation, replication

## Abstract

Avian influenza virus cross-species infection in humans poses a major threat to global public health. Species-specific differences between avian ANP32A and mammalian ANP32 proteins create a natural barrier against viral cross-species infection by directly impairing the functional interaction between the avian-origin viral RNA polymerase and mammalian ANP32 proteins, thereby restricting viral genome replication. The key to overcoming this barrier lies in the adaptation of viral RNA polymerase to host ANP32 family proteins. This mini-review summarizes the mechanisms and variations in influenza virus adaptation to ANP32 proteins across different species. Influenza viruses adapt to species-specific ANP32 proteins through various mutations and display distinct preferences for specific ANP32 family members within the same host. Additionally, alternative splicing variants of ANP32A within a single species further modulate viral RNA polymerase adaptability. Despite this diversity, the underlying interaction mechanism remains conserved: ANP32–polymerase binding is necessary but not sufficient for optimal polymerase activity. This interaction facilitates the formation of asymmetric polymerase dimers and specifically supports viral genome replication, while the step from cRNA to vRNA remains subject to species-specific restrictions. This explains the classic adaptive mechanism of the PB2 E627K mutation, which restores efficient viral genome replication through acid–base pairing with ANP32A. Furthermore, adaptive mutations in emerging strains such as H3N2 canine influenza virus and recent cases of H5N1 in dairy cows underscore the need for continuous viral surveillance and deeper mechanistic studies on virus–ANP32 interactions. Such research is strategically critical for advancing the One Health approach and mitigating future influenza pandemics.

## INTRODUCTION

The influenza virus RNA polymerase is a heterotrimeric complex comprising the PB2, PB1, and PA subunits. It binds to the eight segmented genomic RNAs of the virus, each of which is encapsidated by nucleoprotein (NP) to form viral ribonucleoprotein complexes—the minimal functional replicative units—which together mediate transcription and replication of the viral genome (1).

**Fig 1:**
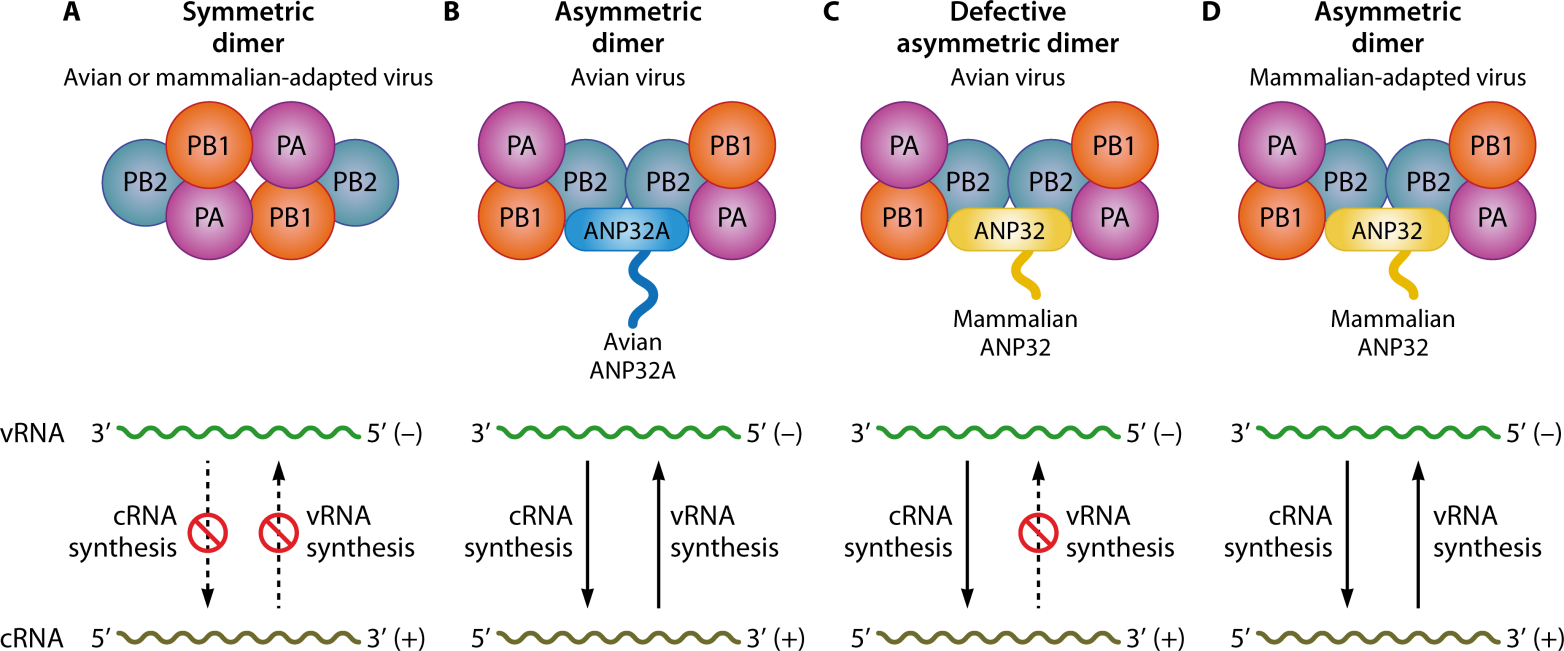
Model depicting the mechanism by which ANP32 proteins regulate influenza virus RNA polymerase activity. (**A**) In cells lacking ANP32A/B, the influenza virus RNA polymerase forms a symmetric dimer and fails to support the synthesis of both cRNA and vRNA. (**B**) Avian ANP32A promotes the formation of an asymmetric polymerase dimer for avian influenza virus (AIV), enabling the synthesis of cRNA and vRNA. (**C**) When the AIV polymerase interacts with mammalian ANP32, it forms an asymmetric dimer that is defective in cRNA—but not vRNA—synthesis. (**D**) Following mammalian adaptive mutations, the avian-origin polymerase associates with mammalian ANP32 to form an asymmetric dimer competent for synthesis of both cRNA and vRNA.

A key host factor regulating this polymerase is the acidic nuclear phosphoprotein 32 kDa (ANP32) family. This family includes several members (ANP32A, ANP32B, and ANP32E) that share a conserved structure: an N-terminal leucine-rich repeat (LRR) domain and a C-terminal low-complexity acidic region (LCAR). These proteins are involved in diverse cellular processes, such as hormone receptor signaling, enzyme inhibition, cell adhesion, intracellular transport, gene expression regulation, and apoptosis (2). Notably, ANP32A, ANP32B, and ANP32E have been specifically implicated in modulating the activity and host adaptability of influenza virus RNA polymerase (3–7). The primary function of ANP32A/B in influenza virus replication is to catalyze the assembly of the viral polymerase into an asymmetric dimer from the symmetric dimer precursor (Fig. 1A, B and D). This asymmetric dimer consists of a replicase (FluPolR) that synthesizes RNA and a partner polymerase (encapsidase, FluPolE), which binds the newly synthesized RNA and assists in its encapsidation into RNPs by recruiting NP (8–11). Thereby, ANP32A/B is essential for enabling the viral polymerase to synthesize both complementary RNA (cRNA) and genomic viral RNA (vRNA) (Fig. 1B and D).

**Fig 2:**
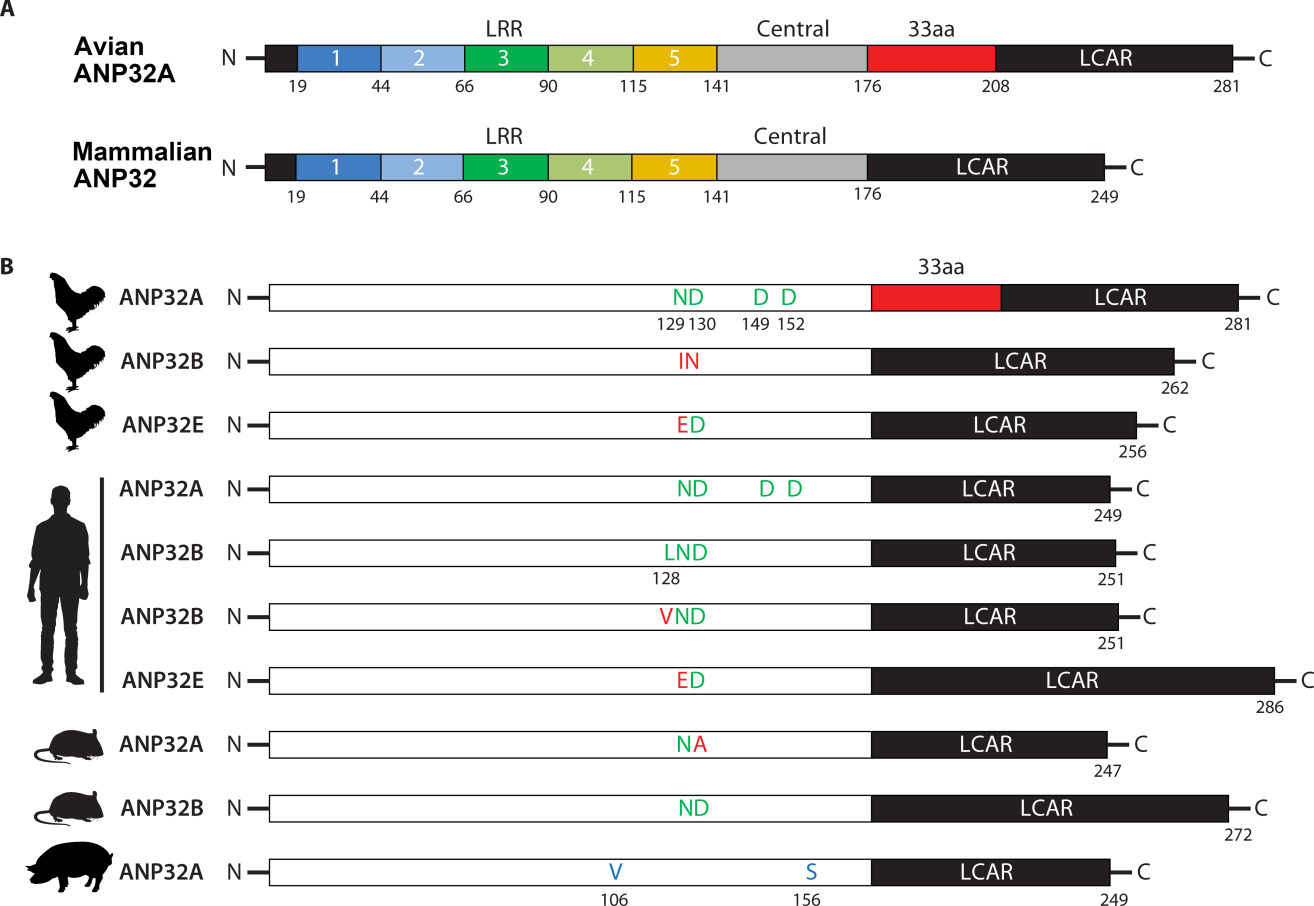
Species-specific differences in avian and mammalian ANP32 proteins and key amino acid (aa) residues influencing influenza A virus (IAV) RNA polymerase activity. (**A**) Structural schematics highlighting species differences. Chicken ANP32A is used to represent the typical structure of avian ANP32A, featuring a 33-aa insertion critical for supporting avian viral RNA polymerase activity. Human ANP32A represents the shorter mammalian ANP32 isoform, which lacks this 33-aa insertion and does not support avian viral RNA polymerase activity. (**B**) Key aa residues on ANP32 proteins identified to affect the interaction with the viral RNA polymerase and its activity. Residues colored green enhance the interaction and polymerase activity, whereas those colored red inhibit the interaction and polymerase activity. Residues colored blue are specific to swine ANP32A and are responsible for supporting the RNA polymerase activity of AIVs.

When AIVs cross species barriers to infect mammalian hosts, multiple stages of their life cycle encounter species-specific restrictions (1). A major hurdle is the functional incompatibility between avian-origin viral RNA polymerase and mammalian ANP32 proteins (3). The species-specific difference between avian ANP32A and mammalian ANP32 proteins (Fig. 2A) creates a particularly severe defect for AIV in one critical step (Fig. 1C): the assembly of a functional FluPolR complex (asymmetric dimer) for the synthesis of vRNA from cRNA. In the defective asymmetric dimer, the RNA-binding site of FluPolE fails to align properly with the product exit channel of FluPolR, resulting in impaired vRNA encapsidation and replication (7, 12, 13). This bottleneck in vRNA production constitutes a major barrier to cross-species transmission (Fig. 1C), which viral adaptive mutations must overcome to establish infection in a new host (Fig. 1D).

## ADAPTABILITY OF INFLUENZA VIRUS RNA POLYMERASE TO ANP32 PROTEINS FROM DIFFERENT SPECIES

In avian cells, ANP32A is the only member of the ANP32 family that effectively supports the activity of AIV RNA polymerase. Avian ANP32B, containing N129I and D130N substitutions, cannot support viral polymerases; consequently, genetic ablation of avian ANP32A abolishes viral polymerase function and prevents genome replication (5, 14). Unlike avian ANP32A, mammalian orthologs (e.g., human, mouse, pig, and dog) lack a 33-aa segment between the N-terminal LRR and C-terminal LCAR domains. This shorter form of mammalian ANP32A fails to support avian influenza polymerase activity, thereby constituting a natural barrier to cross-species transmission (3). To overcome this initial barrier, AIVs can package host-derived avian ANP32A into virions (15). Upon infecting a mammalian cell, this co-delivered protein temporarily boosts early replication by supporting the incoming avian polymerase. Although this aid is transient—as mammalian cells lack avian ANP32A for progeny virions—the increased early viral load critically raises the probability of generating mammalian-adaptive polymerase mutations (e.g., PB2 E627K). Once such mutations arise, the polymerase switches to utilizing the host’s endogenous mammalian ANP32 proteins, enabling sustained infection (15). Adaptation often involves mutations in the viral polymerase that enable compatibility with mammalian ANP32 proteins (Table 1). For example, the PB2 E627K mutation allows polymerases of H5N1, H9N2, and H7N9 viruses to utilize human ANP32A and ANP32B (5). In human cells, single knockout of ANP32A or ANP32B does not impair the polymerase activity of human-adapted viruses; dual knockout is required to abolish activity, indicating functional redundancy between ANP32A and ANP32B in supporting polymerase function (4, 5, 14). Notably, polymerases carrying PB2 E627K exhibit stronger adaptation to human ANP32B than to ANP32A (16). Interestingly, human-adapted IAV polymerases can still use chicken ANP32A (3), but not ANP32B with N129I and D130N substitutions (5, 14). In mice, the polymerase of avian viruses with PB2 E627K relies solely on ANP32B as murine ANP32A carries a residue variation at position 130, which impedes functional support (4, 17). Beyond E627K, other PB2 mutations such as Q591S, D701N, and T473M also facilitate adaptation to mammalian ANP32 in various virus backgrounds like the 2009 pandemic H1N1, H3N2, H7N9, and H9N2 (16, 18). Pigs are considered “mixing vessels” for influenza viruses due to the dual presence of both avian- and human-type sialic acid receptors in their respiratory tract (19), as well as the unique features of porcine ANP32A. aa substitutions at positions 106 and 156 in porcine ANP32A enable weak support of avian viral polymerase activity (20, 21). The 2009 pandemic H1N1 strain uses PB2 Q591R and D701N mutations to adapt to porcine ANP32A/B, with a preference for ANP32A over ANP32B (16). Similarly, H3N2 canine influenza virus (CIV) adapts via PB2 D701N to canine ANP32A, though strains with this mutation remain relatively uncommon (16). Notably, the recently identified PB2-M631L mutation has emerged as a hallmark of H5N1 viruses adapting to bovine hosts, structurally clustering with residues 591 and 627 to modulate interactions with ANP32 (22). Recently, avian viruses with PB2-627V have been shown to efficiently use both avian and human ANP32A, supporting robust replication in chickens and mice. These viruses exhibit dual characteristics: avian-like (possessing the typical E residue at PB2 position 627) and mammalian-like (functionally akin to the PB2 E627K mutation). The emergence of PB2-627V across multiple subtypes may present a public health risk (23).

**TABLE 1:** Summary of key influenza virus adaptive mutations and their interactions with host ANP32 proteins

Host adaptation context	Key adaptive mutation(s)	ANP32 protein preference	Functional outcome	Proposed mechanism
Mammalian (e.g., human and mouse)	PB2 E627K	Human ANP32B > ANP32A; mouse ANP32B (not ANP32A)	Enables use of mammalian ANP32A/B for robust replication; acid-base compatibility	Forms a salt bridge with ANP32A/B E151; compensates for charge incompatibility; enhances binding affinity to human ANP32B/A
Mammalian (e.g., human and mouse)	PB2 T473M (e.g., H7N9 and H9N2)	Human or mouse ANP32A/B (preference unclear)	Synergizes with E627K to boost polymerase activity in human cells	ANP32A LCAR domain interacts with the PB2 region including residue 473, stabilizing the polymerase complex and enhancing replication
Swine (mixing vessel)	PB2 D701N and Q591R (e.g., pdmH1N1)	Porcine ANP32A > ANP32B	Supports efficient polymerase function in pig cells	Q591R forms a salt bridge with ANP32 E151; D701N stabilizes the NLS-627 domain interface; enhances binding to porcine ANP32
Canine	PB2 D701N (e.g., H3N2 CIV)	Canine ANP32A	Supports viral replication in dogs	Alters the nuclear localization signal domain electrostatic surface; fine-tunes polymerase for effective interaction with canine ANP32A
Bovine (emerging)	PB2 M631L (e.g., H5N1 in cattle)	Bovine ANP32 (mechanism under study)	Associated with adaptation and spread in dairy cows	Located within the 627-domain; adjacent to position 627; directly modifies the ANP32-interaction hotspot
ANP32A/B-independent replication in human cells	PB1 K577E and PA Q556R (e.g., H5N1)	ANP32E	Maintains replication in absence of ANP32A/B	PB1 K577E disrupts symmetric dimerization; PA Q556R strengthens interaction with ANP32E; promotes asymmetric dimer formation
ANP32A-independent replication in chicken cells	PB2 D256G, PA T97I, and combinations including PB2 E627K with PA K 339M and M561V (e.g., H5N1)	Unknown (not identified)	Confers replication in absence of avian ANP32A	Mutations rewire host-factor dependency; likely exploit an alternative, unknown host factor
ANP32A-independent replication in gene-edited (GE) chickens	PB2 M631L and PA E349K (e.g., H9N2 and H5N1)	ANP32B and ANP32E	Bypasses requirement for ANP32A; engages ANP32B/E	PA E349K shifts oligomeric equilibrium to asymmetric dimers; PB2 M631L fine-tunes the ANP32-binding interface
Dual avian/mammalian signature	PB2 627V (e.g., H9N2, H7N9, H3N8, and H5N1)	Avian ANP32A and human ANP32A/B	Supports replication in both avian and mammalian hosts; “generalist” strategy	627V provides a plastic interface compatible with both human ANP32A LCAR and chicken ANP32A 33-aa insertion

Under normal conditions, ANP32E does not support influenza polymerase activity. However, serial passaging of IAV A/Turkey/05/2009 (H1N1) (Tky05) was done in human ANP32A/B-knockout cells selected for PB1 K577E and PA Q556R mutations, enabling utilization of ANP32E (6). Similarly, passage of H5 viruses in ANP32A-knockout chicken cells yielded mutant viruses capable of ANP32A-independent replication, harboring mammalian-adaptive mutations such as PB2 D256G, PA T97I, or combinations including PB2 E627K with PA K339M and M561V (24). Although the mechanism remains unclear, these mutations may facilitate compensatory interactions with other ANP32 family proteins. While the generation of chickens with edited ANP32A shows promise as a strategy for curbing avian influenza, high-dose H5 virus challenge can lead to breakthrough infections. This is mediated through adaptive polymerase mutations, such as PA-E349K and PB2-M631L, which enable the virus to co-opt the otherwise less supportive chicken/human ANP32B or ANP32E proteins for replication (25). This plasticity highlights the remarkable adaptive capacity of influenza viruses to exploit alternative ANP32 proteins under selective pressure. Notably, the PB2-M631L mutation, which facilitates viral adaptation in ANP32A-edited chickens, has also emerged in the recent H5N1 strain affecting U.S. dairy cattle as a key adaptation to a new host (22). Long-term use of such poultry requires careful evaluation of potential evolutionary risks, including novel strain emergence and enhanced transmissibility.

Some AIVs enhance mammalian adaptation through nonpolymerase viral proteins. The NS2 (NEP) protein binds SUMO-modified (i.e., post-translationally modified by the Small Ubiquitin-like Modifier) human ANP32A/B via a SUMO-interacting motif, strengthening the ANP32A/B–vRNP interaction and boosting polymerase activity, thereby facilitating adaptation to human hosts (26). Additionally, ANP32 proteins interact directly with NP via the LCAR domain, facilitating the recruitment of NP to replication complexes and promoting packaging of newly synthesized RNA into progeny RNPs (9).

Furthermore, species-specific differences in ANP32A not only restrict avian-to-human transmission of IAVs but also limit the activity of human influenza B virus (IBV) polymerases in avian cells (27). Intriguingly, IBV polymerase is able to use murine ANP32A, which is incapable of recovering IAV polymerase activity (4).

Overall, influenza viruses employ diverse key mutations to adapt to ANP32 proteins across species and exhibit distinct preferences among ANP32 family members within a host, significantly complicating efforts to control cross-species transmission.

## DIFFERENTIAL ADAPTABILITY OF INFLUENZA VIRUS RNA POLYMERASE TO ANP32A SPLICE VARIANTS WITHIN A SINGLE SPECIES

Multiple alternative splice variants of ANP32A are expressed within individual species. Owing to differences in their aa sequences, these variants exhibit distinct capacities to regulate AIV RNA polymerase activity, thereby directly influencing viral replication efficiency in the host. In avian species, three principal transcript variants of chicken ANP32A have been identified—designated ANP32A-X1 (ANP32A₃₃), ANP32A-X2 (ANP32A₂₉), and ANP32A-X3—which differ substantially in their ability to support the replication of influenza viruses, particularly AIVs (28). Compared to ANP32A-X1, the ANP32A-X2 isoform lacks four critical amino acid residues (VYSE, residues 129–132), significantly impairing its capacity to support polymerase activity in viruses carrying the avian-characteristic PB2-627E (28–30). ANP32A-X3, which shares greater sequence homology with mammalian ANP32A, fails to support AIV RNA polymerase function (28). Further analysis indicates that ANP32A-X1 represents the most abundant functional variant in chicken and duck cells (28, 29). This distribution critically influences the inherent susceptibility of avian cells to AIV infection. However, the skewed expression pattern of ANP32A splice variants in some avian species—characterized by lower ANP32A-X1 (swallow and magpie) and elevated ANP32A-X3 levels (goose and swan)—may promote the selection and persistence of mammalian-adapted influenza viruses (28). Notably, the splicing regulator SRSF10 promotes the alternative splicing of chicken ANP32A, shifting the expression from the 33-aa form (ANP32A-X1) to a 29-aa variant (ANP32A-X2). This shift correlates with direct inhibition of AIV polymerase activity. By reducing the abundance of functional ANP32A-X1, SRSF10-mediated splicing modulation represents a novel host mechanism to balance viral replication, fine-tuned through the regulation of ANP32A isoform expression (31). Similarly, equine ANP32A (eqANP32A) undergoes alternative splicing, generating six documented variants with varying expression levels. These isoforms differentially support the RNA polymerase activity of equine influenza virus. Among them, eqANP32A_X2 is not only the most highly expressed but also the most effective in supporting polymerase function (32). These findings suggest that the relative abundance of specific ANP32 splice variants within a cell can modulate its susceptibility to influenza viruses. Consequently, research into the regulatory mechanisms governing ANP32 alternative splicing may yield novel strategies and therapeutic targets for the control and prevention of influenza virus infections.

## MECHANISMS OF ANP32 REGULATION OF INFLUENZA VIRUS RNA POLYMERASE ACTIVITY AND HOST ADAPTABILITY

### Interaction between ANP32 and influenza virus RNA polymerase

The interaction between ANP32 proteins and the influenza virus RNA polymerase is essential for polymerase activity (Fig. 2). Residues L128, N129, and D130 in ANP32A/B are directly involved in binding the viral polymerase (5, 14, 33). Mutations at polymerase sites that interact with these residues—such as PA K413, PA D529, and PB2 T609—impair the ANP32A–polymerase interaction and reduce RNA synthesis (8). Introduction of D149Y and D152H mutations into chicken or human ANP32A also reduces binding to the polymerase, inhibiting both polymerase activity and viral replication (34). Porcine ANP32A contains I106V and P156S substitutions that enhance its binding to avian influenza polymerase and facilitate viral replication in pigs (20, 21). Chicken ANP32A contains a 33-aa insertion not found in mammalian orthologs, which strengthens its interaction with both avian- (PB2-627E) and human-adapted (PB2-627K) polymerases. Nevertheless, it enhances the activity of the avian-virus polymerase to a much greater extent than that of the mammalian-adapted polymerase (35, 36), suggesting that the 33-aa segment influences polymerase function beyond mere binding. These indicate that the polymerase–ANP32 interaction is necessary but not sufficient for optimal activity. The PB2 E627K mutation enables avian polymerases to use human ANP32 proteins, where the positively charged lysine forms a critical salt bridge with E151 in the acidic loop of human ANP32A/B. Initial studies suggested this was not due to strengthened binding to human ANP32A (35, 36), but more recent work utilizing sensitive techniques has revealed that human ANP32A binds to PB2-627K via multiple low-affinity interactions involving its C-terminal LCAR, which are weaker and less abundant with the avian-adapted 627E (37). The PB2 Q591R substitution functions analogously to E627K by forming a salt bridge with residue E151 of ANP32, thereby supporting robust viral replication in human cells in the absence of E627K (10, 11). The interface between the NLS and 627 domains of PB2 is the proposed binding interface of the host ANP32 LCAR domain. The PB2 D701N mutation substitutes a neutral asparagine (N) for a negatively charged aspartate (D) at position 701. This alters the electrostatic surface of the NLS domain, making it more complementary for binding the acidic, negatively charged LCAR of human ANP32 (10). During the 2009 pandemic, D701N and Q591R acted cooperatively to optimize the polymerase complex for high-affinity interaction with porcine ANP32 (16). Similarly, in canine H3N2 influenza, the PB2 D701N mutation fine-tunes the polymerase for effective binding to canine ANP32A, facilitating sustained transmission in dogs (16). Found in the recent bovine H5N1 outbreak, the PB2 M631L mutation resides within the 627-domain, adjacent to position 627 (22). It directly fine-tunes the binding interface between the PB2 627-domain and the ANP32 LCAR, thereby facilitating viral adaptation in mammalian hosts (10, 11). Furthermore, in contexts where human ANP32A/B are limiting, the PA Q556R mutation can enhance viral replication by strengthening the interaction with mammalian ANP32E proteins to support robust polymerase activity (6, 10). Together, these mutations demonstrate a multifaceted strategy where direct engagement of ANP32 (via E151 and LCAR) is synergistically combined with allosteric modulation of polymerase stability and oligomerization to achieve host adaptation. In IBV, species-specific differences in ANP32A limit both interaction and polymerase activity of human strains in avian cells (27).

### ANP32 regulates viral replication and imposes mammalian restriction on cRNA-to-vRNA synthesis

The influenza virus polymerase transcribes viral mRNA and replicates the genomic RNA. Replication occurs in two steps: vRNA-to-cRNA and cRNA-to-vRNA synthesis. In mammalian hosts, ANP32A and ANP32B specifically support both replication steps for adapted viral polymerases, but do not affect transcription (7, 12, 13). In contrast, avian ANP32A supports the complete replication cycle (both steps) for avian virus (12). Structurally distinct from avian ANP32A, mammalian ANP32 proteins restrict the activity of avian influenza polymerases. Consequently, avian viruses can use human ANP32A/B for cRNA synthesis but not for vRNA production (13, 29); this impairment is specific to the cRNA-to-vRNA synthesis step. Thus, species-specific differences in ANP32A regulate the cRNA-to-vRNA synthesis step during influenza genome replication. Furthermore, in ANP32A/B-knockout cells, PB1 K577E and PA Q556R mutations allow the viral polymerase to use ANP32E, enhancing vRNA synthesis (6).

### ANP32 mediates asymmetric dimerization of the viral polymerase

Cryo-EM structures of IAV, IBV, and influenza C virus polymerases in complex with ANP32 proteins demonstrate that ANP32A facilitates the assembly of an asymmetric dimer—consisting of one FluPolR and one FluPolE—essential for viral genome replication, despite notable structural variations among different virus types (8, 10, 11). In influenza C virus, ANP32A stabilizes FluPolR and induces a conformational change in FluPolE, reorienting the C-terminal domain of PB2 (including the PB2-627 and PB2-NLS domains) toward the C-terminus of the PA (P3) subunit (8). In IAV, structures of H5N1 polymerase bound to human ANP32B reveal a similar asymmetric dimer, where the PB2-Mid-link and PB2-CBD domains of FluPolE interact with the PB2-N2 domain of FluPolR (11). This reorientation aligns the RNA-binding site of FluPolE with the product exit channel of FluPolR, enabling coordinated replication and encapsidation (8, 11). In contrast to ANP32A, which binds both FluPolE and FluPolR of influenza C virus (8), ANP32B in IAV specifically interacts only with FluPolE. This binding is mediated by the C-terminal LRR region of ANP32B and involves direct contact with the PB2 627-domain (11). Acting as an electrostatic chaperone, the host factor ANP32 binds and stabilizes the free, unengaged viral polymerase (apo-polymerase), facilitating the formation of a polymerase dimer (10). One subunit of this ANP32-stabilized dimer is subsequently delivered to an RNP-associated replicase to assemble the functional asymmetric replication dimer (10, 11). However, recent structural studies reveal a key functional divergence in this process between influenza types. In IBV, ANP32 directly pre-forms and stabilizes the FluPolE conformation within the symmetric apo-polymerase dimer (10). In stark contrast, for IAV, while ANP32 binds and solubilizes the apo-polymerase, the complex remains in a transitional state. The stable, mature FluPolE architecture is only achieved upon its delivery to the RNP-associated replicase, where it is cooperatively defined within the final asymmetric replication complex (10, 11). Thus, ANP32 acts as a pre-forming chaperone for FluB, but primarily as an escort and bridging factor for FluA.

In the absence of ANP32A and ANP32B, the viral polymerase assembles into symmetric dimers. This defective structure disrupts the proper alignment between the RNA-binding site of FluPolE and the product exit channel of FluPolR, which is essential for both RNA protection and subsequent encapsidation (6, 11). While these symmetric dimers retain some basal replication activity (38), they are functionally suboptimal compared to the ANP32-stabilized asymmetric dimers. However, compensatory mutations such as PB1 K577E and PA Q556R restore viral replication in ANP32A/B-knockout cells by facilitating asymmetric dimerization through recruitment of ANP32E (6). The PB1 K577E mutation functions by weakening the symmetric dimer (FluPolA) interface. In contrast, the PA Q556R mutation in the FluPolE promotes the formation of the asymmetric dimer. This promotion is achieved through the formation of a salt bridge with E154, a residue conserved across ANP32A, B, and E proteins (6, 10, 11). When influenza virus replicates in transgenic chickens or chicken embryos with edited, nonfunctional ANP32A, viral populations are dominated by the PA E349K and PB2 M631L mutations, which enable the utilization of both chicken and human ANP32B and ANP32E (25). The PA E349K mutation acts indirectly by shifting the polymerase’s oligomeric equilibrium towards the replication-competent asymmetric dimer, while the PB2 M631L mutation likely enhances the interaction of the FluPolE with suboptimal ANP32 proteins (ANP32B and ANP32E) (10, 11), further promoting the formation of the polymerase asymmetric dimer and thereby facilitating replication. Collectively, mutations that disrupt the symmetric dimer interface, combined with those enhancing ANP32 binding, cooperatively promote the formation of the asymmetric dimer and restore the activity of the influenza viral RNA polymerase. Structural data also indicate that the endonuclease active site of FluPolR is occluded, consistent with its inability to perform transcription (8, 10).

### Acid–base properties of PB2-627 and adaptation to ANP32

In the asymmetric polymerase dimer, the acidic LCAR domain of ANP32 (ANP32Aⁿᶜᴬᴿ) inserts between the PB2-627 domains of the two polymerase subunits. Human ANP32Aⁿᶜᴬᴿ contains exclusively negatively charged acidic residues, favoring interaction with basic (e.g., positively charged lysine, K) or neutral (e.g., uncharged valine, V) residues at PB2-627. In contrast, chicken ANP32Aⁿᶜᴬᴿ contains an additional 33-aa segment rich in both acidic and basic residues, enabling it to support polymerases with acidic (E), basic (K), or neutral (V) residues at PB2-627 (8). The neutral valine at position 627 confers compatibility with both avian and human ANP32 proteins (23). ANP32 proteins function as electrostatic chaperones, recruited to the viral polymerase through long-range attractive forces (e.g., nonspecific electrostatic interactions) (10), where precise acid–base complementarity at the ANP32–PB2 interface then stabilizes the asymmetric dimer and enhances the polymerase activity. These indicate that polymerase–ANP32 interaction with precise electrostatic compatibility is crucial for optimal activity.

## PERSPECTIVE

While this review has focused on the adaptation of AIVs to mammalian hosts through ANP32 proteins, studying the evolution of influenza viruses in other established mammalian reservoirs is crucial for a comprehensive understanding of pandemic risk. A key example is the H3N2 CIV. Since its emergence, H3N2 CIV has accumulated mutations adaptive to humans during circulation in dogs, leading to enhanced polymerase activity in human cells. The PB1 D154G mutation is a key marker of human adaptation in CIV (39), distinct from mutations found in avian-origin viruses such as H5N1 and H7N9. Whether PB1 D154G or other mutations mediate adaptation to human ANP32 proteins and what risk it poses for human cross-species infection remain open questions. The recent spillover of H5N1 into dairy cows and other mammals like farm cats (40), coupled with its high transmissibility in lactating goats (41), underscores the urgent need to investigate influenza virus adaptation through host factors such as ANP32 proteins. Continuous surveillance and mechanistic studies of viral adaptation in emerging hosts are essential for assessing public health risks and implementing precise control measures.

The mechanism by which influenza viruses adapt to ANP32 proteins across species appears conserved: ANP32 binding promotes asymmetric dimer formation and facilitates cRNA-to-vRNA replication. However, viruses employ distinct mutations to adapt to different mammalian ANP32 proteins and exhibit clear preferences among ANP32 family members within a host. The structural basis for these preferences remains unclear. Furthermore, the potential for viruses adapted to one mammalian species to infect others requires further evaluation. Future studies using artificial intelligence to analyze structural interactions between mutant polymerases and ANP32 proteins may reveal why different mutations are required in different hosts, whether functional convergence occurs, and how adaptation to one host affects compatibility with ANP32 proteins from others. Such insights will inform the design of broad-spectrum antivirals and improve the prediction of viral evolution and cross-species transmission risk.
